# Magnetic Investigation of Cladded Nuclear Reactor Blocks

**DOI:** 10.3390/ma15041425

**Published:** 2022-02-15

**Authors:** Gábor Vértesy, Antal Gasparics, Ildikó Szenthe, Sándor Bilicz

**Affiliations:** 1 Centre for Energy Research, Institute of Technical Physics and Materials Science, 1121 Budapest, Hungary; gasparics.antal@ek-cer.hu (A.G.); szenthe.ildiko@ek-cer.hu (I.S.); 2 Department of Broadband Infocommunications and Electromagnetic Theory, Faculty of Electrical Engineering and Informatics, Budapest University of Technology and Economics, Műegyetem rkp. 3., H-1111 Budapest, Hungary; bilicz.sandor@vik.bme.hu

**Keywords:** magnetic nondestructive evaluation, nuclear reactor pressure vessel, austenitic cladding, steel degradation

## Abstract

The wall, made of ferromagnetic steel, of a nuclear reactor pressure vessel is covered by an austenitic (very weakly ferromagnetic) cladding. In this work, we investigated how the base material and the cladding can be inspected separately from each other by nondestructive magnetic measurements. It was found that with the proper choice of the magnetizing yoke, these two different materials could be measured independently of each other. The effect of the yoke’s size was studied by the numerical simulation of magnetic flux, pumped into the material during magnetic measurements. Measurements were performed by two different sizes of yokes on pure base material, on base material under cladding and on cladding itself. Experiments verified the results of the simulation. Our results can help for the future practical application of magnetic methods in the regular inspection of nuclear power plants.

## 1. Introduction

In almost all major industrial countries worldwide today, nuclear power plants (NPPs) are used to generate electricity. However, the Fukushima Daiichi accident affected the nuclear energy renaissance and, since then, safety aspects have been significantly strengthened. For many existing NPPs, lifetime extensions to 40, 50, 60 or even 80 years have been requested [1]. The long-term operation of existing NPPs has already been accepted in many countries as a strategic objective to ensure adequate supply of electricity over the coming decades. Operating conditions that affect the design lifetime include neutron exposure (fluence), as well as the number and magnitude of temperature and/or pressure cycles in both normal conditions and hypothetical accidental conditions [2,3]. License renewal and periodic safety reviews (PSRs) are the two basic regulatory approaches that are required for an authorization of the long-term operation of NPPs [1]. Evaluation of these parameters during the PSRs allows for an estimation of the operational lifetime of NPPs [2].

As a result of the above arguments, the regular inspection of nuclear power plants is an extremely important task, because the mechanical properties of the reactor pressure vessel (RPV) wall are modified during its operation mainly due to the long-term and high-energy neutron irradiation [4]. In boiling water pressurized reactors, the most critical and most important part is the reactor pressure vessel, because it is not changeable during the whole period of operation. Apart from the standard destructive tests (mechanical Charpy impact testing [5]) several nondestructive electromagnetic methods have been suggested recently for determination of neutron irradiation-generated embrittlement of the pressure vessel steel material. The precise measurement of the Seebeck coefficient makes it possible to derive the neutron irradiation-induced embrittlement of RPV material [6,7]. An ultrasonic technique is also widely used in the inspection of NPPs [8,9,10]. 

The RPV material is ferromagnetic steel, so magnetic methods are useful for this inspection. An overview of nondestructive magnetic methods are given in References [11,12]. The magnetic Barkhausen noise (MBN) technique was developed for inspection of residual stresses, surface defects and microstructure changes [13,14,15,16]. Another method, magneto-acoustic emission, can also be frequently used for the monitoring of residual stresses [17]. 

A correlation exists between the modification of the microstructure of the material, generated by different effects, and the observed magnetic behavior if the material is influenced by a magnetic field. This phenomenon can be used to characterize the ferromagnetic materials via magnetic hysteresis measurement. Dislocation movement and domain wall motion are both affected by the microstructure of the material. In ferromagnetic materials, the correlation between mechanical and magnetic hardness is well-known and understood [18,19]. Magnetic methods are advantageous because they are not expensive, are technically simple, and they can be used easily, even on active materials. One of them, the so-called 3MA approach (3MA = micromagnetic, multiparameter, microstructure and stress analysis) applies several methods [20,21,22], and it was found suitable for the characterization of the damage in ferromagnetic materials like RPV steels and for monitoring the progress of materials. 

Another method of magnetic nondestructive testing is the measurement of the magnetic hysteresis loops. For instance, the so-called magnetic minor loops power scaling laws (PSL) were developed, whereby different parameters of minor hysteresis loops are used for material characterization [23]. A similar method, magnetic adaptive testing, also measures systematically minor magnetic hysteresis loops. This is also a multi-parametric, powerful and sensitive method of magnetic inspection [24]. As a conclusion of these works, a reasonable correlation could be found between the destructively measured parameters and nondestructively measured magnetic characteristics through application of these methods. This fact makes possible the future potential use of magnetic methods in the inspection of RPVs’ structural integrity.

In several previous works [16,20,25,26,27] irradiated Charpy samples were measured using magnetic methods, and results of magnetic measurements were compared with the destructively measured ductile to brittle transition temperature (DBTT). However, for the full inspection of nuclear reactors, blocks cut from RPV steel should be also measured, not only samples of Charpy geometry. This aspect of the inspection of a nuclear reactor’s integrity has still not been extensively investigated. If the base material itself is directly measured, magnetic measurement is easy. However, in RPV, the base material (ferromagnetic steel) is covered by a cladding. This cladding, which is made of austenitic steel, is the integral component of a WWER 440-type nuclear reactor pressure vessel. Its role is to warrant an anticorrosive protection for the vessel material. Cladding is about a 10 mm thick stainless steel weld-overlay, which is deposited on the pressure vessel’s inner surface. It shields the base metal of the pressure vessel from the corrosive environment produced by primary light water coolant [28]. Cladding in WWER 440 reactors is made by submerged arc welding technology by using strip electrodes. The surface of the cladding is either ground or machined roughly to ensure ultrasonic testing coupling.

For future inspection of nuclear reactors, a tool should be developed that is suitable for non-destructive evaluation of the embrittlement of the vessel wall. The final system should be capable of inspecting the degradation of the microstructure through the cladding. The first step has been made in this direction: cladded blocks were successfully measured even through the cladding [29] via the magnetic adaptive testing method [24]. In this work, cladded RPV blocks were investigated, which had been treated thermally by a step cooling procedure, which caused embrittlement of the material. It was demonstrated that the base material degradation could be followed by magnetic measurements even through the cladding. It was shown that a reliable, nearly linear correlation existed between magnetic parameters and DBTT, as expected.

In this type of investigation, when base material is measured through the cladding, the main problem for the magnetic measurement is that cladding means a thick, almost nonmagnetic layer between the magnetizing yoke and base material, which resulted in an extremely low and noisy probe response, as presented in Reference [29]. In other words, cladding causes serious difficulty for base material investigation. Nevertheless, through the proper choice of measuring parameters and suitable software, the measured signal could be successfully evaluated.

On the other hand, the impact of cladding on the reactor pressure vessel wall integrity has been investigated in a very limited way in spite of the fact that it can potentially be of great significance to RPV integrity. The reason is that plasticity and the elevated fracture toughness of cladding can provide additional strength to the pressure wall and this process can justify an extended reactor lifetime [30]. In addition, in thermal expansion coefficients, significant differences can be found with respect to pressure vessel base metals, which can cause a stress peak [31]. This is the so-called pressurized thermal shock and it is a potential risk of interfacial crack initiation and propagation. Safety analysis of this phenomenon has lately become a subject of interest for operators of nuclear power plants [32]. In a very recent work, the results of an experimental investigation were presented, aimed at the evaluation of microstructure and failure mechanisms of WWER 440 reactor pressure vessel austenitic cladding (made of stainless steel Sv 08Kh19N10G2B) [33]. 

The question is arising, as to whether the base material and cladding could be investigated independently of each other by magnetic measurements. The purpose of this paper was to study this problem and to give an answer to this question. Material of cladding is basically austenitic, but it also contains several percentages (2–8%) of ferrite (magnetic) phase. The existence of this ferrite phase gives a chance for successfully applying magnetic measurements to study the properties of cladding. However, the huge volume of highly ferromagnetic base metal, close to the weakly ferromagnetic cladding material, causes difficulty for cladding material investigation. In this work, we will show that the characterization of base and cladding material can be separated from each other by a suitable technique of measurement.

The idea is to choose the proper size of magnetizing yoke—different sizes of yokes can be used for the characterization of base metal and for the characterization of cladding. To make a qualitative interpretation of the experimental results, the effect of the yoke dimension is calculated by numerical simulation of the magnetic flux distribution in the sample. 

## 2. Materials and Methods

### 2.1. Materials 

**Figure 1:**
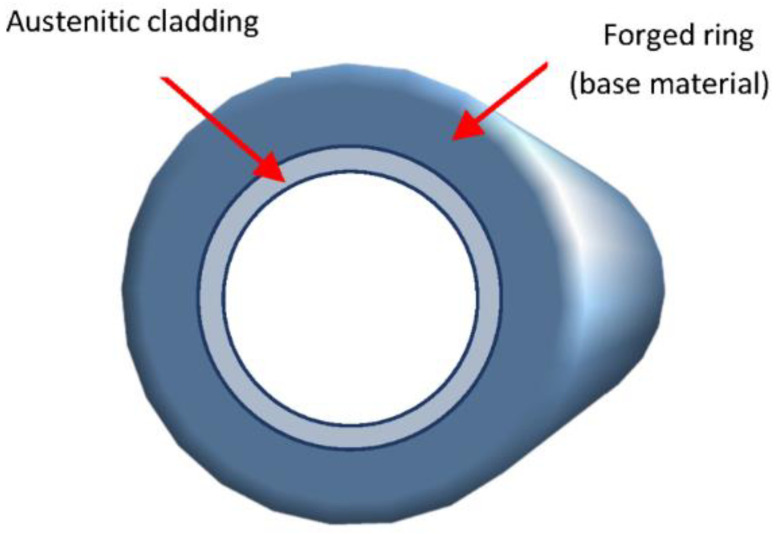
Cutting of the cladded block from a forged ring.

**Figure 2:**
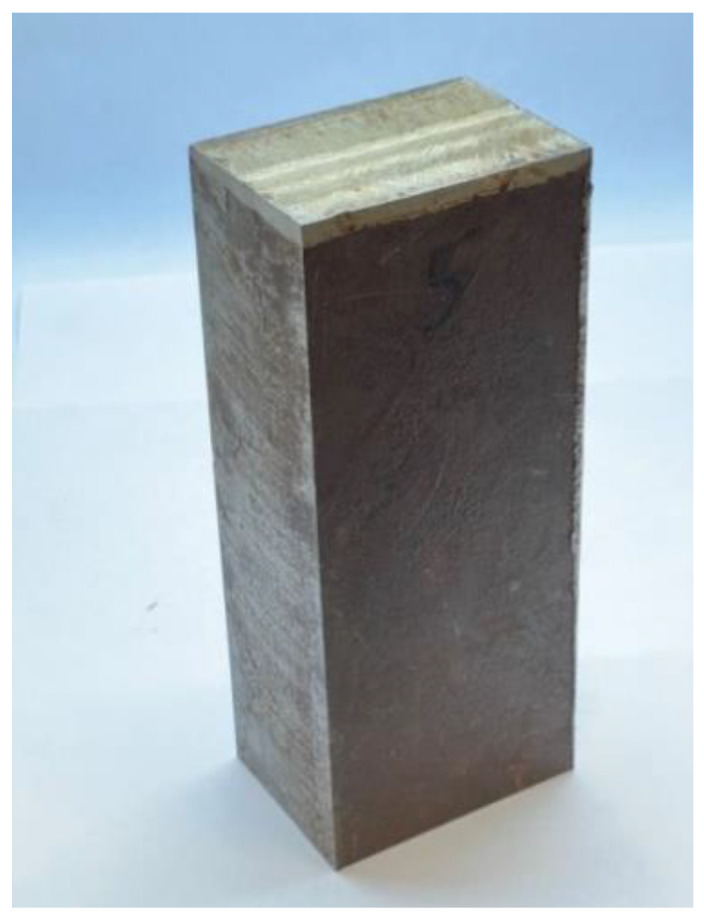
Photograph of the cladded block.

As a first sample, a cladded block was investigated. Chemical composition of the 15H2NMFA base material can be seen in Table 1. The block was cut from the forged ring according to Figure 1. This block is shown in Figure 2. The size of the block was 110 mm × 77 mm × 278 mm. The 10 mm thick cladding is clearly seen on the top of the block. 

**Figure 3:**
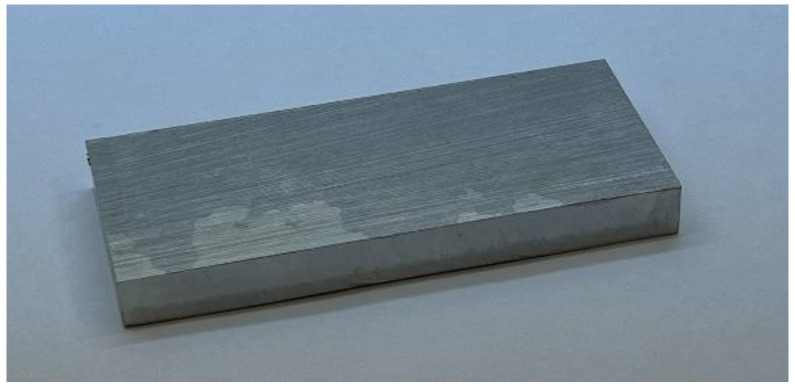
Photograph of the cladding itself (cut from the top of another cladded block).

Another sample was also prepared. This was only a piece of cladding, which was cut from the top of a cladded block. The photograph of this sample is shown in Figure 3. The size of the sample was 114 mm × 50 mm × 12 mm.

### 2.2. Magnetic Measurement

Permeability of the material was measured by attaching a magnetizing yoke on the surface of the sample. The yoke itself was made of Fe-Si laminated sheets. An exciting coil, a wound on the leg of magnetizing yoke, was used for producing magnetizing field *F* in the sample. 

Before starting the measurement, the sample was magnetized close to saturation by applying a magnetizing current in the exciting coil. Then, the value of magnetizing current was decreased, linearly by time *t*, to zero and then increased again in the opposite direction up to saturation with the same slope. The slope of the magnetizing current was 0.125 A/sec. Due to the time variation of the effective field in the magnetizing circuit, a signal is generated in the pick-up coil, which is wounded also on the magnetizing yoke. As long as the magnetic field (or magnetizing current) is sweeping linearly with time, *t*, the *U* signal voltage in the pick-up coil is proportional to the differential permeability, *μ* of the magnetic circuit.

*μ* = const × *U(*d*F*/d*t) =* const × ∂*B(*d*F*/d*t)*/∂*t* = const × *μ(*d*F*/d*t) ×* d*F*/d*t* (1)

In future parts of the text, magnetizing current, *I*, will be used instead of magnetizing field to describe the magnetization of the sample, because in an open magnetic circle the real value of magnetizing field inside the sample is never known due to the dissipation of the magnetic field into the air. It means that in non-uniform magnetic circuits, it is not possible to speak about the signal *U* as proportional to the differential permeability of the material, but we use an effective differential permeability values of the existing circuit. The current values also characterize the magnetic state of the investigated samples well. 

**Figure 4:**
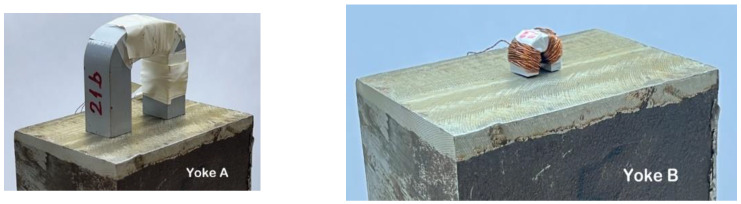
Photographs of two different size magnetizing yokes (**Yoke A** and **Yoke B**) on the top of the same block.

The magnetizing yokes with different dimensions, Yoke A and Yoke B, used in our measurements, can be seen in Figure 4. The two (driving and pick-up) coils, wound on the legs of yokes, are seen well in the photos, especially on the right side photo (Yoke B). 

The signal of the pick-up coil can be seen well in figures below in Research and Discussion section. The magnetizing current value at the maximal value of permeability was used as the characteristic parameter for the magnetic behavior of the investigated samples. During measurements, the steel side of the block (down in Figure 2), the cladded side of the block (up in Figure 2) and the cladding (Figure 3) were measured by applying two different size magnetizing yokes. 

**Figure 5:**
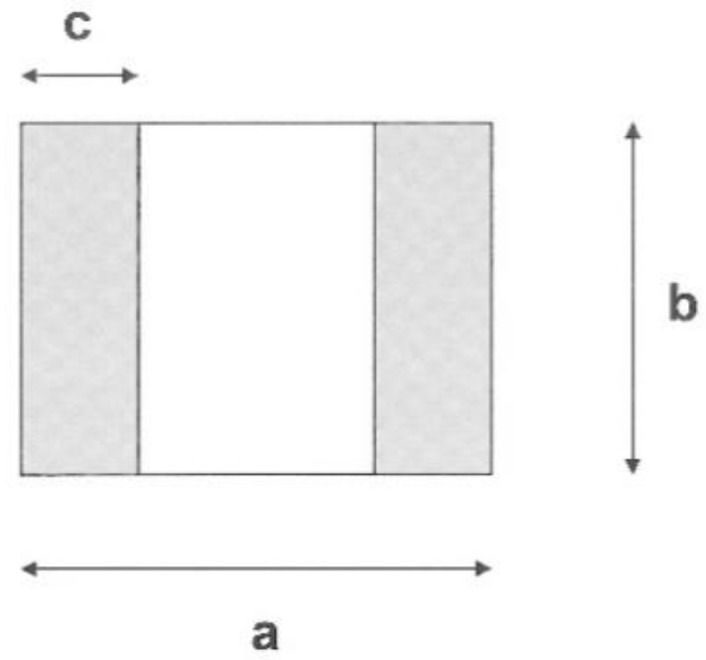
Schematic drawing of the sole of magnetizing yoke. a: total length of the yoke, b: width of the yoke, c: width of the leg.

The schematic drawing of the sole of magnetizing yoke is given in Figure 5, and Table 2 presents the dimensions of the two yokes numerically. In this table, the heights of the yokes are also given.

### 2.3. Numerical Simulation

The idea behind applying two different sizes of magnetizing yokes for measurement of cladded blocks was that by doing this, we can separate the magnetic signal from the base material and from the cladding. Numerical simulation of the distribution of the magnetic flux was performed for both Yoke A and Yoke B. The ferromagnetic base material was characterized by a nonlinear *B*(*H*) curve with saturation around 2 T and initial relative permeability of *μ_rel,0_* = 1200. Calculations were performed for three different values of relative permeability of the cladding, *μ_rel_* = 1, 5 and 10, respectively.

The calculations have been performed by 3D Finite Element Method (FEM), using the COMSOL Multiphysics software. The partial differential equations of the stationary magnetic field have been formulated for the magnetic vector potential [34]. The model domain was closed by an artificial boundary on which the normal component of the magnetic flux density was set to zero. The exciting coil has been modelled as an equivalent surface current density on the yoke’s surface. The coil on Yoke A has 150 turns, whereas Yoke B has 40 turns. The exciting current was set as 0.45 A and 0.3 A, respectively, which approximated the exciting current at maximum differential permeability in the experiments. The nonlinear system of equations resulting from the FEM-discretization has been solved iteratively by the software.

In the post-processing step, two magnetic fluxes were calculated: Ψ_1_ is the flux together in the cladding and in the base material, while Ψ_2_ is the flux only in the base material, both evaluated at the symmetry plane of the model. The quotient Ψ_2_/Ψ_1_ characterizes the relative magnetization of the base material. The numerical results for the two yokes and three different values of the relative permeability of the cladding, are given in Table 3.

It can be seen that in the case of Yoke A, this was a large value (>93%), while in the case of Yoke B, it was only around 50%. In this latter case, Ψ_2_/Ψ_1_ depended more on the relative permeability of the cladding. 

**Figure 6:**
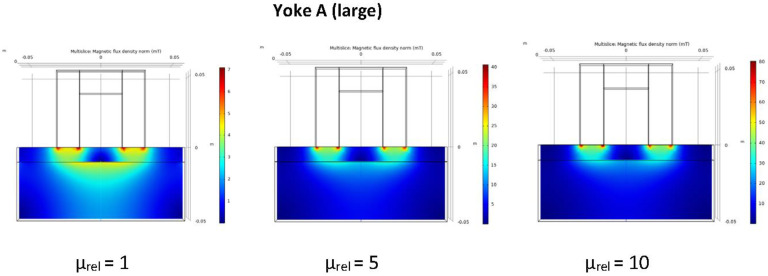
Distribution of the calculated flux density in the cladded block for three values of relative permeability of the cladding if the large magnetizing yoke (Yoke A) is applied.

**Figure 7:**
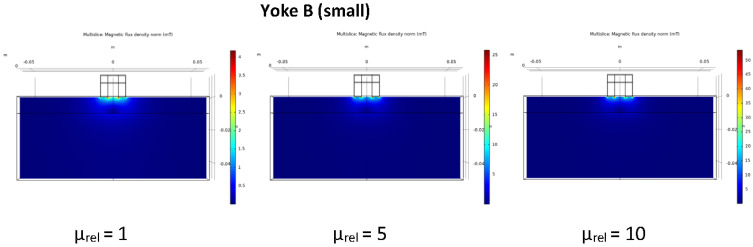
Distribution of the calculated flux density in the cladded block for three values of relative permeability of the cladding if the small magnetizing yoke (Yoke B) is applied.

The distribution of the magnetic flux is shown for the three values of the relative permeability of cladding (*μ_rel_* = 1, 5 and 10) for both yokes in Figure 6 and Figure 7.

The result of numerical simulation revealed that by using the large magnetizing yoke, the base material could be magnetized enough even through the cladding. On the other hand, if a small-sized magnetizing yoke is applied, it is sensitive only to the region of cladding, while the magnetic influence of the base material below cladding is very limited, almost negligible. In the next section, it will be shown how the real measurements verified the result of simulation.

## 3. Results and Discussion

### 3.1. Yoke A

**Figure 8:**
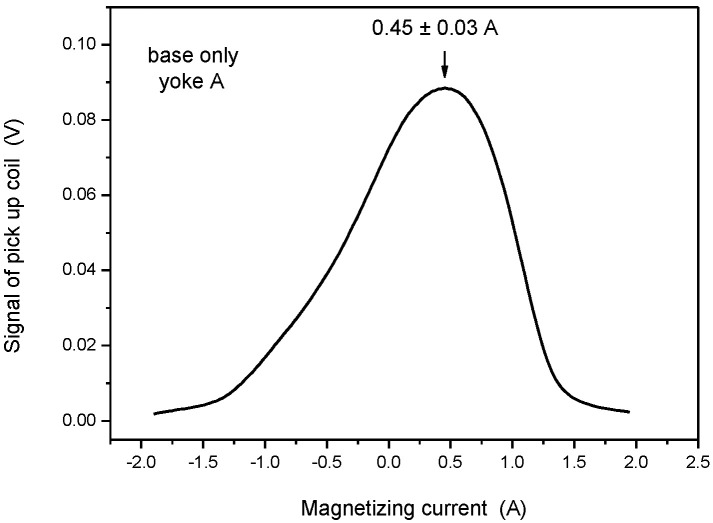
Signal of the pick-up coil as a function of the magnetizing current, measured on base material by applying Yoke A.

The first measurement was performed on the base material (bottom of block, shown in Figure 2), by applying the larger yoke (Yoke A). The signal of the pick-up coil (proportional to the permeability of the material according to Equation (1)) as a function of the magnetizing current is presented in Figure 8. The error of the magnetizing current at the top permeability is also given in the figure. The sample magnetically was saturated before measurement by a negative current, then the value of the magnetizing current was linearly decreased to zero, then increased with the same slope of current to positive saturation. 

The value of the magnetizing current at the maximal value of permeability was chosen to characterize the magnetic behavior of the measured material. This value, which was 0.45 A in the case shown above, does not depend on the actual parameters of the measurement. Evidently, only results of those measurements can be compared with each other, which were performed by the same magnetizing yoke. 

**Figure 9:**
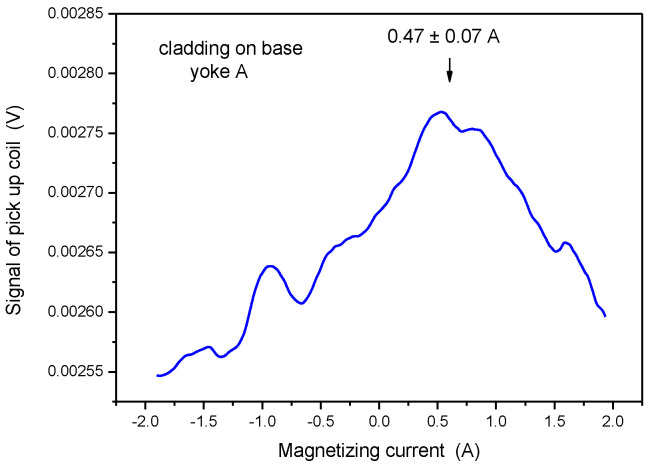
Signal of the pick-up coil as a function of the magnetizing current, measured on the top of cladded block by applying Yoke A.

The next measurement was performed on the top of the block (shown in in Figure 2), again by applying Yoke A. In this case, cladding and base material were measured together. In principle, the magnetic behavior of both somehow influence the measured signal. However, as concluded from the result of simulation, a relatively high amount of flux was pumped into the ferromagnetic base material, so it is expected that mainly the high permeability base material determines the measured signal. The result of this measurement is shown in Figure 9. The values of the magnetizing currents at maximal permeability were very close to each other (0.45 A and 0.47 A), within the error of measurement. This means that base material was also detected when measurement is performed through the cladding. The influence of cladding was almost negligible in this case. This result verifies our previous measurements on thermally treated cladded blocks [31].

It should be mentioned, however, that the registered curve is rather noisy if measurement is performed through cladding. For better presentation, the curve of Figure 9 was smoothed. Smoothing, made by adjacent averaging of measured points, decreases the scatter of points, but it has no influence on the value of magnetizing current at the maximal permeability. Measurements were repeated five times after each other, removing and placing the magnetizing yoke back. Practically no difference was found in the registered curves by this repetition of measurement. 

**Figure 10:**
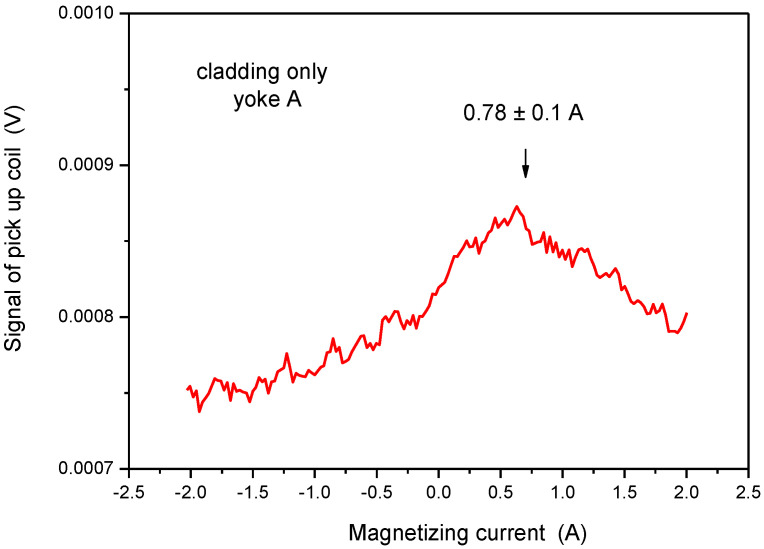
Signal of the pick-up coil as a function of the magnetizing current, measured on the pure cladding by applying Yoke A.

**Figure 11:**
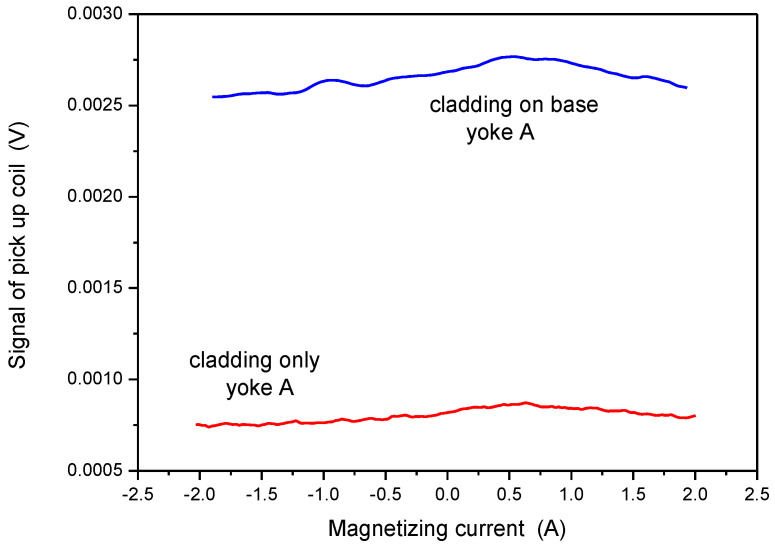
Signal of the pick-up coil as a function of the magnetizing current, measured on the pure cladding and on the cladding above the base material by applying Yoke A.

In order to study the situation better, measurement by Yoke A was done also on pure cladding (see sample in Figure 3). This result is shown in Figure 10. In accordance with our expectation, a weak maximum can be seen in permeability, but at different values of magnetizing current, compared to measurements made either on base material or on cladding above base material. The maximum of the curve appeared at I = 0.47 A if the base material was under the cladding and appeared at I = 0.78 A in the case of pure cladding. This difference cannot be explained by any experimental error, only by the difference in the magnetic behavior of the cladding and base material. The magnetic behavior of cladding is due to the small ferrite content of cladding, as mentioned already in the Introduction. The signal was very low and noisy, but a maximum definitely existed. The two signals, measured on pure cladding and on the cladding above the base material, can be compared if the two curves are presented on the same scale, as done in Figure 11.

### 3.2. Yoke B

**Figure 12:**
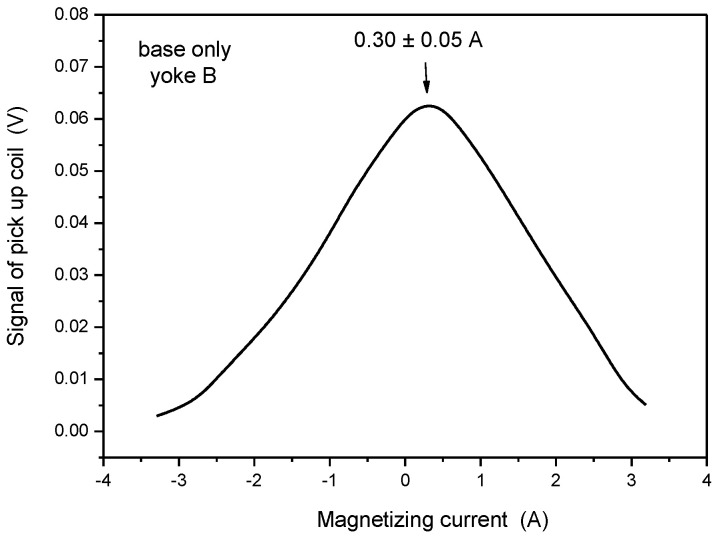
Signal of the pick-up coil as a function of the magnetizing current, measured on the base material by applying Yoke B.

**Figure 13:**
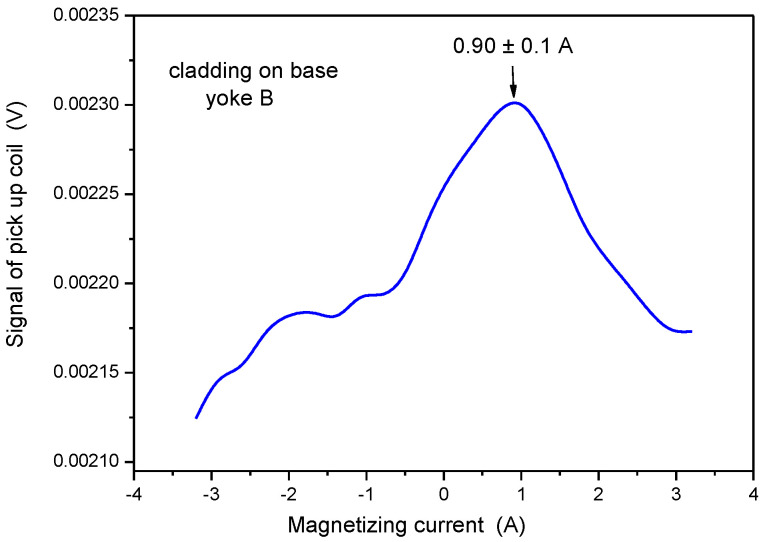
Signal of the pick-up coil as a function of the magnetizing current, measured on the top of the cladded block by applying Yoke B.

**Figure 14:**
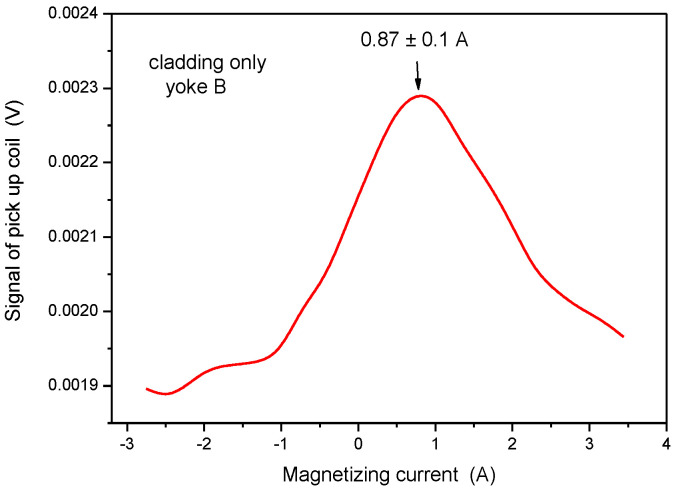
Signal of the pick-up coil as a function of the magnetizing current, measured on the pure cladding by applying Yoke B.

The same series of measurements as described above was performed on the samples by applying the small-sized yoke. Results are shown in Figure 12, Figure 13 and Figure 14. Note that the values of magnetizing current at maximal permeability are not comparable with similar current values in the previous section because different yokes were used in the two measurements. 

As defined above, the characteristic parameter of the base material was 0.30 A (see Figure 12). When the same measurement was performed, but on the cladding, the magnetizing current at maximal permeability was 0.90 A (see Figure 13). This parameter was very far from the base metal, so it can be considered as characteristic for the cladding. This statement is confirmed by the result of the measurement, performed on pure cladding (see Figure 14), where the maximum was observed at 0.87 A magnetizing current. 

When the small size yoke was applied, the influence of the base material could not be detected, but the magnetic behavior of cladding could be excellently measured, even in the presence of highly ferromagnetic base metal. 

Application of magnetic nondestructive methods can be important for the future inspection of austenitic steel degradation. As is known, originally paramagnetic steel became more and more ferromagnetic under stress, due to the appearance of bcc α’-martensite. In our previous work, titanium stabilized austenitic stainless steel, 18/8 type, was studied [35]. Stainless steel specimens were cold-rolled at room temperature. The compressive plastic deformation of the material increased its hardness. It was found that this change could be followed by a nondestructive magnetic method with substantially higher sensitivity and reliability than the traditionally used destructive hardness measurements. 

In another work, austenitic stainless steel SUS316L was also plastically deformed by a tensile stress [36]. In contrast to the compressed samples, the tensile deformation did not introduce such a large percentage of the ferromagnetic phase into the deformed samples. Nevertheless, magnetic indication of the strain values was possible, and the method was also able to reflect anisotropy induced into the material by the stress.

## 4. Conclusions

A magnetic method was developed by which the cladded blocks of a nuclear pressure vessel can be characterized by a nondestructive technique. 

It was demonstrated, by applying two magnetizing yokes with different dimensions, that the two types of very magnetically different components (ferromagnetic base metal and almost austenitic cladding) can be investigated separately from each other. To our best knowledge, this way of measurement is new. 

If a large-sized yoke is applied, the ferromagnetic base metal can be measured. This yoke is not suitable for investigation of cladding on the top of base material, because the signal from the ferromagnetic part suppresses the signal of weakly magnetic cladding. 

With the application of a small-sized yoke, the cladding itself can be measured, even in the presence of highly ferromagnetic steel. The magnetic flux is closed in the cladding and cannot penetrate into the base material. 

The effect of the different yoke dimension was determined based on simulation of the magnetic flux distribution in the given geometry. The results of measurement correlate very well with the suggestions of simulation.

By using our results, the possible material degradation of austenitic cladding on the pressure vessel—due to different effects, like neutron irradiation, thermal treatment, etc.—can be inspected by a simple and nondestructive magnetic method. This way of investigation implies the ability to monitor the integrity of the various layers of the reactor walls. Furthermore, this approach of using two different yoke sizes to test a bilayer material can be applied in general in other areas, where a highly ferromagnetic material is covered by another weakly ferromagnetic layer. This would make the work more meaningful than in connection with the testing of one particular wall. This seems to be possible, since the depth of magnetic permeability testing should increase with the size of the yoke.

Based on our results, in the future, it will be possible that material degradation of cladding generated by any effects (neutron irradiation, thermal shock, plastic or elastic deformation) could be inspected by magnetic hysteresis measurements, mainly by magnetic adaptive testing. The measurement can even be done directly on the reactor pressure vessel wall. 

## Figures and Tables

**Table 1:** Chemical composition of 15H2NMFA base metal (wt%).

C%	Mn%	Si%	S%	P%	Cr%	Ni%	Mo%	V%	Cu%	Co%	Sb%	Sn	As%
0.16	0.42	0.29	0.08	0.012	1.97	1.29	0.52	0.12	0.12	0.06	0.001	0.003	0.003

**Table 2:** Dimensions of the magnetizing yokes.

	a (mm)	b (mm)	c (mm)	Height (mm)
Yoke A	62.0	19.0	16.0	55.0
Yoke B	11.5	12.5	4.5	13.0

**Table 3:** Numerical result of simulation of the magnetic flux for the two different yokes and for three different values of relative permeability of the cladding.

	*μ_rel_*	Ψ_1_ (Wb)	Ψ_2_ (Wb)	Ψ_2_/Ψ_1_
Yoke A	1	4.55 × 10^–6^	4.24 × 10^–6^	0.931
Yoke A	5	1.49 × 10^–5^	1.43 × 10^–5^	0.956
Yoke A	10	2.54 × 10^–5^	2.43 × 10^–5^	0.955
Yoke B	1	3.35 × 10^–7^	1.96 × 10^–7^	0.586
Yoke B	5	1.12 × 10^–6^	5.88 × 10^–7^	0.526
Yoke B	10	1.95 × 10^–6^	9.86 × 10^–7^	0.505

## Data Availability

The data are contained within the article.
